# Comprehensive Quantitative Analysis of SQ Injection Using Multiple Chromatographic Technologies

**DOI:** 10.3390/molecules21081092

**Published:** 2016-08-19

**Authors:** Siu-Leung Chau, Zhi-Bing Huang, Yan-Gang Song, Rui-Qi Yue, Alan Ho, Chao-Zhan Lin, Wen-Hua Huang, Quan-Bin Han

**Affiliations:** 1 School of Chinese Medicine, Hong Kong Baptist University, Hong Kong, China; dumdum1986@msn.com (S.-L.C.); rickyyue@126.com (R.-Q.Y.); alanhhm@hkbu.edu.hk (A.H.); 2 State Key Laboratory of Food Science and Technology, Sino-Germany Joint Research Institute, Nanchang University, Nanchang 330047, China; hzbchem@ncu.edu.cn; 3 Limin Pharmaceutical Factory, Livzon Group, Shaoguang 512028, China; Songyangang@livzon.cn; 4 Institute of Clinical Pharmacology, Guangzhou University of Traditional Chinese Medicine, Guangzhou 510405, China; linchaozhan@gzucm.edu.cn

**Keywords:** Shenqi Fuzheng injection, Chinese medicine formula, quantitative analysis, chemical profile

## Abstract

Quality control of Chinese medicine injections remains a challenge due to our poor knowledge of their complex chemical profile. This study aims to investigate the chemical composition of one of the best-selling injections, Shenqi Fuzheng (SQ) injection (SQI), via a full component quantitative analysis. A total of 15 representative small molecular components of SQI were simultaneously determined using ultra-high performance liquid chromatography (UHPLC) coupled with quadrupole tandem time-of-flight mass spectrometry (Q-TOF-MS); saccharide composition of SQI was also quantitatively determined by high performance liquid chromatography (HPLC) with evaporative light scattering detector (ELSD) on an amino column before and after acid hydrolysis. The existence of polysaccharides was also examined on a gel permeation chromatography column. The method was well validated in terms of linearity, sensitivity, precision, accuracy and stability, and was successfully applied to analyze 13 SQI samples. The results demonstrate that up to 94.69% (*w*/*w*) of this injection product are quantitatively determined, in which small molecules and monosaccharide/sucrose account for 0.18%–0.21%, and 53.49%–58.2%, respectively. The quantitative information contributes to accumulating scientific evidence to better understand the therapy efficacy and safety of complex Chinese medicine injections.

## 1. Introduction

Quality control of herbal injection remains a challenge. Different from traditional Chinese patent drugs, herbal injection has a very short history. Born in the army hospital during war time [1], the first herbal injection (bupleurum injection) was initially created for the heavily injured soldiers when penicillin was severely short of supply. As both a great invention and a crazy risk, herbal injection has received a great deal of recognition and criticism over the past decades. Especially in recent years, many herbal injection products have been licensed and marketed, whereas many medicine-injury incidents regarding adverse reactions caused by herbal injection have also been reported. Compared to other drug preparations, injection has more strict criteria for quality control. Furthermore, the complicated chemical profile of herb origins renders herbal injections a special safety concern. Qualitative and quantitative knowledges about the chemical profile of herbal injections is the basis for improving the quality control of herbal injections; however, information in these aspects is quite limited.

In our previous studies, we have qualitatively and quantitatively analyzed different preparations of herbal medicines, such as the Huang Lian Jie Du decoction and Shuang-huang-lian oral solution [1,2]. Not only small molecules, but also sugars and polysaccharides were quantitatively determined in these Chinese patent drugs. The full component analysis approach quantitatively revealed up to 78% (*w*/*w*) of the chemical ingredients in these samples. We wish to quantitatively explore the chemical profile of herbal injections, taking Shenqi Fuzheng injection (SQI) as an example due to its importance.

Since approved and marketed in China as an antitumor injection in 1999 [3], Shenqi Fuzheng injection, which contains two herb components, Radix Astragali and Radix Codonopsis, has been one of the best-selling herbal injections in China. It has raised attention to its significant efficacy in clinical treatment, such as in the therapy in intractable heart failure [4], gastric cancer [5,6], colorectal cancer [7], lung cancer [8], and breast cancer [9,10]. It is popularly used in combination with chemotherapy because of its use in treating side effects of chemotherapy [11,12,13,14,15]. It was also reported to have protective effects on diabetic glomerulopathy [16] and immune function enhancement [17,18], as well as therapeutic potential of viral myocarditis [19] in animal experiments. Although it is known that alkaloids, isoflavones, saponins, amino acids, and saccharides [20,21,22,23,24,25,26] are the ingredients of two herbal components of the Shenqi Fuzheng injection, only two chemicals, calycosin-7-*O*-α-glucoside and astragaloside IV, have been quantitatively determined in this injection [27].

Due to conflicts of interest, Shenqi Fuzheng injection samples were not directly available. Alternatively, its manufacturer provided some lab-made samples named SQ injection. This study aims to establish a full component analysis to quantify both the small molecules and saccharides of SQ injection, using ultra-high performance liquid chromatography (UHPLC) coupled with quadrupole tandem time-of-flight mass spectrometry (Q-TOF-MS), and high performance liquid chromatography equipped with evaporative light scattering detector (HPLC-ELSD). The existence of polymers was also examined by using high performance gel permeation chromatography (HPGPC).

## 2. Results and Discussion

### 2.1. Qualitative Analysis of Constituents

**Figure 1:**
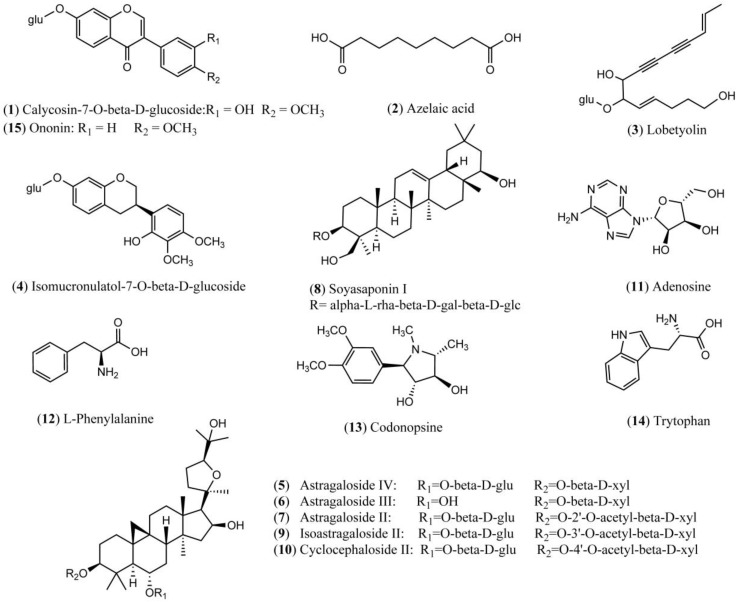
Chemical structures of 15 identified non-sugar small molecules.

**Figure 2:**
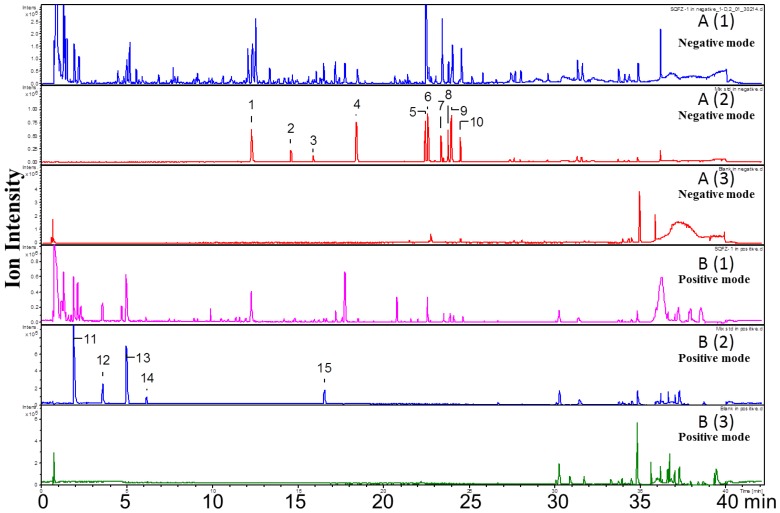
UHPLC-Q-Tof-MS chromatogram of SQI samples (A1 and B1), reference standards (A2 and B2), and blank samples (A3 and B3) in negative and positive ion mode.

**Figure 3:**
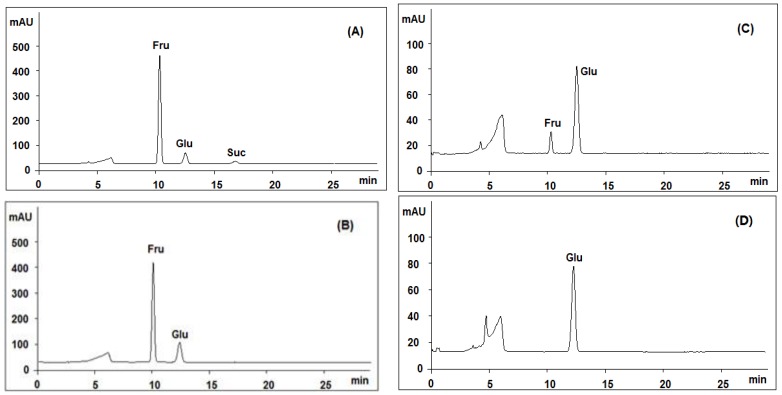
Comparative analysis of the products generated by acid hydrolysis of SQI sample at different concentrations of trifluoroacetic acid (TFA): (**A**) 0 M; (**B**) 0.02 M; (**C**) 1 M; (**D**) 2 M; using HPLC-ELSD coupled with an Asahipak Amino P-50 4E column.

Both positive and negative ion modes were chosen for MS analysis depending on the different chemical properties of analytes. As shown in Figure 1, 15 representative non-sugar small molecules in total were unambiguously determined by comparing retention times (Figure 2), accurate *m*/*z* and fragmentation data with reference standards (Table 1). They included three isoflavones (calycosin-7-*O*-β-glucoside, ononin, isomucronulatol-7-*O*-β-d-glucoside), five saponins (astragaloside IV, astragaloside III, astragaloside II, isoastragaloside II, and soyasaponin I), and a glycoside (cyclocephaloside II) from Radix Astragali, an alkaloid (codonopsine) and a polyacetylene (lobetyolin) from Radix Codonopsis, and two amino acids (trytophan, l-phenylalanine), a dicarboxylic acid (azelaic acid), as well as a purine nucleoside (adenosine) [20,21,22,23,24,25,26]. The saccharide profile was also shown in Figure 3, in which only three saccharides—fructose, glucose and sucrose—were identified by comparison with reference standards.

**Figure 4:**
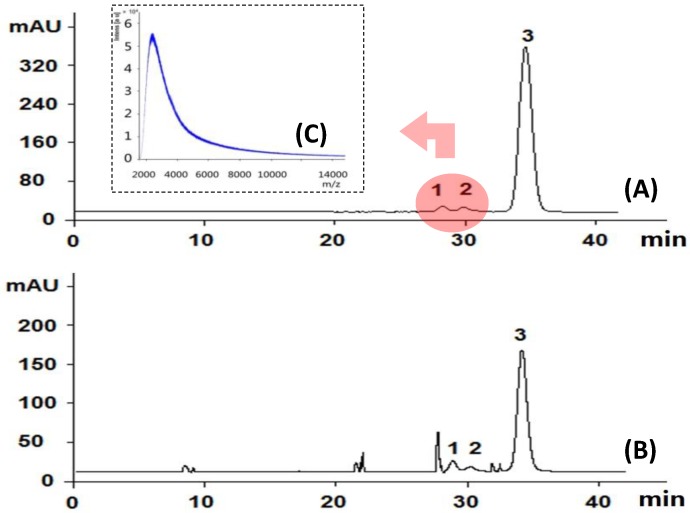
HPGPC analysis of SQI sample before (**A**) and after (**B**) acid hydrolysis; MALDI-TOF-MS analysis of peaks 1 and 2 (**C**) The determined small molecules contribute peak 3.

HPGPC is popularly used to determine the homogeneity of purified polysaccharides in carbohydrate polymer research. It is also recently used to profile the molecular size distribution pattern of tested samples [28,29,30]. The HPGPC analysis of SQI, as shown in Figure 4A, indicated three peaks at 28.5 min (peak 1), 29.3 min (peak 2), and 35 min (peak 3).

According to our previously work [28,29,30], the major peak No. 3 was comprised of small molecules, and those two small peaks (peaks 1 and 2) around 28–30 min were supposed to be polymers, possibly polysaccharides. In order to determine if they are polysaccharides, we compared the monosugar profile and HPGPC chromatogram before/after acid hydrolysis. If they are polysaccharides, they are supposed to be hydrolyzed to monosugars, so that peaks 1 and 2 should disappear and the content of monosugars should increase. As shown in Figure 4B, peaks 1 and 2 remain unchanged after acid hydrolysis, even at the strict condition (2 M TFA in 120 °C for 2 h) which is usually used for complete acid hydrolysis of polysaccharides [31]. Furthermore, neither the content of glucose nor that of total monosaccharide increased after acid hydrolysis at varied TFA concentrations, as shown in Figure 3B–D. These results suggested that these two peaks should not be polysaccharides. Although both herbal components of SQI are rich in polysaccharides, SQI should not contain these polymers due to the special operation of ethanol precipitation. These two peaks were further isolated using HPGPC and were subsequently analyzed by MALDI-TOF-MS (Figure 4C). The results showed that the molecular weight of these polymers is around 2000 Da. They were proposed to be some stabilizing agents added in injection preparation.

### 2.2. Limitation of Qualitative Analysis Solely Using Mass Spectrometry

Mass spectrometry has been applied for quantitative and also qualitative analysis in many fields, especially in Chinese medicine analysis. Although it is a powerful and sensitive analytical method as it can provide an accurate mass of the molecules and nanogram of detection limit, solely using MS data in qualitative analysis may bring incorrect identification results. For example, geniposide had been identified in SQI samples solely based on the MS data in some published studies [25], but it could not be found in our samples by comparing both the retention time and ion fragmentation pattern with chemical reference standard. And among these chemicals, codonopsine as the representative marker of Radix Codonopsis, is found as a major ingredient of SQI for the first time. It is suggested that the qualitative analysis only using ion fragmentation information is risky. Moreover, the ionization mode is an important factor for MS analysis. The chemical diversity of Chinese medicines requires different ionization modes. Some components may be ignored if the single ionization mode is used. The comparison between two modes is necessary, which can avoid missing information.

### 2.3. Method Validation

The linearity, regression and linear ranges of 15 analytes are shown in Table 2. The data exhibited a satisfactory relationship between concentrations and peak areas of the analytes within the test ranges (*R*^2^ ≥ 0.9980). The overall RSDs of intra- and inter-day variations for 15 analytes were not beyond 3.74% and 4.81%, respectively. The limits of quantification (LOQ) and limits of detection (LOD) of all analytes were less than 1.06 and 0.41 µg on column, respectively. The established method demonstrated acceptable accuracy with spike recovery of 94.97%–106.59% for all analytes; and the RSDs of the peak areas for 15 analytes detected within 24 h were lower than 4.73%. These results suggested that the developed UPLC-MS method was accurate and reliable for simultaneous quantitative determination of the 15 investigated compounds in SQI injection.

### 2.4. Quantification of Eighteen Analytes in Commercial Shenqi Fuzheng Injection (SQI) Samples

The results of quantitative analysis are summarized in Table 3. In general, the total content of known chemical components reached 88.13%–94.69% of the dry weight of SQI samples. Among them, 15 representative non-sugar small molecules possessed 0.18%–0.21%, and monosaccharide/sucrose accounted for 53.49%–58.2%. In addition, according to the published preparation protocol, three salts, NaCl, edetate disodium, and sodium pyrosulfite, were added at the fixed concentration of 7.2 mg/mL, respectively [32,33,34]. The additives including the stabilizing agent may contribute the majority of the undetermined remainder. These results exhibited a general feature of SQI′s chemical profile: saccharides and salts are the major components, and the non-sugar small molecules possess a very low content.

Safety is often a serious concern in Chinese medicines, especially injection products. Decoction is safer because the digestion system provides protection. In order to make the injection safer, people removed macromolecules such as proteins and polysaccharides from the water decoction using ethanol precipitation. Although the content of small molecules relatively increased, the total dosage of small molecules in injection is much lower than decoction. The safety of SQI is attributed to its special chemical composition. Firstly, both herb materials of SQI are edible. Secondly, the majority (over 90%) of the composition is made up of saccharides/salts and other additives which are all safe and approved materials. Although some of the non-sugar small molecules were undetermined, the determined chemicals represented almost all types of the chemical ingredients of SQI. More importantly, their content is not very high.

On the other hand, the relatively low content of small molecules does not mean that SQI would be inactive. First of all, with a special method of administration, injection does not need a dosage of chemicals as high as that of water decoction. In contrast, a very high dose of small molecules may bring a safety concern. Second of all, SQI contributes a sufficient dosage of these bioactive small molecules, because its recommended dosage is 250 mL/day; in other words, 10 mg/day of these 15 determined non-sugar small molecules is a composition comparable to that of other Chinese medicine injections, e.g., Shenfu and Shengmai injections [35,36]. In a published report [25], 81 chemicals in total were identified in SQI using UPLC/MS, and 32 of them were confirmed with reference standards. It is suggested that the actual intake of these bioactive small molecules should be significantly more than 10 mg/day. Third of all, as found in this study for the first time, alkaloids which usually have potent bioactivity are one of the major ingredients of SQI. For example, adenosine contributes the largest amount to SQI, around 2 mg/day, among all the 15 determined constituents.

The active ingredient profile which is responsible for the therapy efficacy of SQI has not been determined. For example, astragaloside are often thought to be one of the active ingredients of Radix Astragali. However, our previous study indicated the high-quality large roots contain significantly less saponin than the poor-quality small root ends, due to their low ratio of bark where many more saponins exist. It is suggested that saponins should not be the sole quality control (QC) marker of Radix Astragali. Furthermore, glucose is normally the major saccharide, but SQI contains a large amount of fructose (around 35%, mainly from Radix Codonopsis), which is quite different from other Chinese formulae. It is not yet known whether fructose contributes to the therapy efficacy of SQI, and alkaloids also deserve further exploration. The accumulation of evidence of the chemical-activity relationship will be helpful for quality control of Chinese medicine injections.

## 3. Materials and Methods

### 3.1. Chemicals and Materials

Acetonitrile (UPLC grade) was purchased from Baker Analyzed Ltd. (Center Valley, PA, USA), and HPLC-grade acetonitrile, methanol, and formic acid were purchased from Merck (Darmstadt, Germany). Deionized water was prepared by Millipore Milli Q-Plus system (Millipore, Bedford, MA, USA). The reference compounds (Table 2), calycosin-7-*O*-β-d-glucopyranoside (lot No. 130225), astragaloside III (130225), astragaloside IV (131206), adenosine (130225), astragaloside II (130224) and soyasaponin I (130430) were purchased from Chemstrong Scientific Co., Ltd. (Shenzhen, China). Lobetyolin (MUST-13012901), cyclocephaloside II (MUST-13042617) and isoastragaloside II (MUST-13042509) were purchased from Chengdu Must Bio-Technology Co., Ltd. (Chengdu, China). Trytophan (SM0313GE14), (−)-phenylalanine (SM0305RA14) and azelaic acid (SM0313GF14) were purchased from Shanghai Yuanye Bio-technology Co., Ltd. (Shanghai, China). Isomucronulatol-7-*O*-β-d-glucoside (131021) and ononin (130524) were purchased from Victory Bio-technology Co., Ltd. (Sicheung, China). Codonopsine was provided by Dr. Chaozhan Lin, Guangzhou University of Chinese Medicine (Guangzhou, China). Reference substances of d-(−)-fructose, d-(+)-glucose and sucrose were purchased from Sigma-Aldrich (St. Louis, MO, USA). The purity of each standard compound was determined to be higher than 98% by using UPLC-MS.

The raw materials of Radix Astragali and Radix Codonopsis were identified as the dried roots of *Astragalus membranaceus* var. *mongholicus* and *Codonopsis pilosula* by Dr. CAO Hui, respectively. The herb extract and the SQI samples were prepared for research purpose by the research laboratory of Livzon Pharmaceutical Group Inc. (Table 3) using published methods [30,31,32]. In brief, these two herbal materials (40 g each) were extracted separately with boiling water; the water decoctions were then condensed and precipitated in ethanol solution before the supernatants were combined. The combined extract solution was filtered repeatedly with injection-use carbon and diluted to 1000 mL. Three salts, NaCl, edetate disodium, and sodium pyrosulfite, were added at the fixed concentration of 7.2 mg/mL, respectively [32,33,34].

### 3.2. Sample Preparation

Standard solutions of these 15 reference compounds were prepared in water/methanol at the known concentration (mg/mL): adenosine (1.46), phenylalanine (1.47), tryptophan (1.57), codonopsine (0.76), calycosin-7-*O*-β-d-glucopyranoside (0.97), azelaic acid (1.05), lobetyolin (1.22), isomucronulatol-7-*O*-β-d-glucoside (1.10), astragaloside IV (0.64), astragaloside III (0.74), astragaloside II (0.57), soyasaponin I (0.83), isoastragaloside II (0.59), cyclocephaloside II (0.76) and ononin (1.40). All standard solutions were stored at 4 °C until used. The sample solution was filtered through a 0.22 µm nylon-membrane filter (Millipore, Barcelona, Spain) before analysis. The sample (4 mL) was freeze dried and weighed to obtain the total chemical content of the injection samples.

### 3.3. Ultra-Performance Liquid-Chromatography Tandem Mass Spectrometry (UPLC-MS) Conditions

An Acquity ultra-performance liquid chromatography (UPLC) system consisting of an auto-sampler, a binary pump and a PDA detector (Waters, Milford, MA, USA) was used. The compounds were separated on an Acquity BEH C18 (2.1 mm × 100 mm, 1.7 µm; Waters, Milford, MA, USA) analytical column coupled with a guard column (2.1 mm × 5 mm, 1.7 μm). The column and auto sampler were maintained at 40 °C and 10 °C, respectively. A gradient elution was performed using 0.1% (*v*/*v*) formic acid in water (A) and 0.1% (*v*/*v*) formic acid in acetonitrile (B) at a flow rate of 0.35 mL/min, in the following gradient program: 0–2 min, 5% B; 2–18 min, 5%–25% B; 25–33 min, 25%–75% B; 33-36 min, 75%–100% B; 36–39 min, 100% B. A volume of 1 µL was injected in the system for negative mode and 8 µL for positive mode analysis, respectively.

MS data were obtained on a Bruker MicroTOF-Q (Bruker Daltonics GmbH, Bremen, German, quadrupole-time-of-flight (Q-TOF) mass spectrometer with electrospray (ESI) ion source. Operating parameters in the negative and positive ion mode were as follows: nebulizing gas (N_2_) flow rate, 8.0 L/min; nebulizing pressure, 2.0 bars in negative mode and 2.5 bars in positive mode; drying gas temperature, 180 °C; capillary voltage, 4 kV in negative mode and 4.5 kV in positive mode. Mass spectra were recorded across the range *m*/*z* 100–1700 in negative and 50–1600 in positive mode.

The 15 analytes were simultaneously measured in positive mode (adenosine, phenylalanine, trytophan, codonopsine and ononin), and negative mode (calycosin-7-*O*-β-d-glucopyranoside, azelaic acid, lobetyolin, isomucronulatol-7-*O*-β-d-glucoside, astragaloside IV, astragaloside III, astragaloside II, soyasaponin I, isoastragaloside II and cyclocephaloside II) due to their specific response in mass spectrometry.

### 3.4. HPLC-NH_2_P-ELSD Conditions

A previously reported HPLC-NH_2_P-ELSD method was used here to determine the monosaccharides and oligosaccharides in SQI samples [2]. Briefly, an Agilent 1100 liquid chromatography system (Agilent Technologies, Palo Alto, CA, USA) and Alltech 2000 evaporative light scattering detector (Grace Alltech, Deerfield, IL, USA) coupled with an Asahipak Amino P-50 4E (4.6 mm × 20 mm, Shodex, Tokyo, Japan) column at 30 °C were used. A gradient elution was achieved using water (A) and acetonitrile (B) at a flow rate of 0.8 mL/min. The gradient program was used according to the following profile: 0–16 min, 78% B; 16–20 min, 78%–62% B; 20–30 min, 62%–60% B; the drift tube temperature of ELSD was set at 120 °C and the nitrogen flow rate of ELSD was set at 3.2 L/min. The gain number was equal to 1.

### 3.5. High Performance Gel Permeation Chromatography (HPGPC) Conditions

The macromolecules in SQI samples were examined using HPGPC on an Agilent 1100 series HPLC system (Agilent Technologies, Palo Alto, CA, USA) coupled with evaporative light scattering detector (ELSD). The separation was achieved on a two tandem TSK GMPW_XL_ columns (300 mm × 7.8 mm i.d., 10 μm) system operated at 40 °C. Ammonium acetate aqueous solution (20 mM) was used as mobile phase at a flow rate of 0.6 mL/min. The signal from ELSD was transmitted to an Agilent Chemstation for processing through an Agilent 35900E interface. The parameters of ELSD were set as follows: the drift tube temperature was 120 °C; nebulizer nitrogen gas flow rate was at 3.2 L/min; impact off mode. An aliquot of 20 μL solution was injected for analysis.

### 3.6. Acid Hydrolysis

Every sample of SQI (5 mL) was freeze-dried (Labconco, Kansas City, MO, USA, 7400 series). The lyophilized powder was hydrolyzed in 5 mL of TFA solution at varied concentrations (0 M, 0.02 M, 1 M and 2 M) in 120 °C for 2 h to release component monosaccharides. After the hydrolysis, the solvent was removed using a vacuum rotary evaporator, and the remaining TFA was expelled by adding 5 mL methanol to form volatilizable ester in a vacuum rotary evaporator. The dried hydrolysis product was re-dissolved in 5 mL ultrapure water, and filtered through a 0.22 μm Polytetrafluoroethylene (PTFE) syringe filter before HPLC analysis.

### 3.7. Method Validation

The method for quantitative analysis was validated according to the linearity, sensitivity, precision, accuracy and stability performance. A total of 15 reference compounds were diluted with water to appropriate concentrations for forming calibration curves. At least eight concentrations and duplicates are needed; followed by plotting the peak areas versus the concentration of each analyte. Aliquots of the diluted solutions were analyzed by UPLC-MS. The limits of detection (LODs) and limits of quantification (LOQs) under the present chromatographic conditions were determined at a signal-to-noise ratio (*S*/*N*) of about 3 and 10, respectively. The precision was carried out by intra-day variations determination. For the intra-day variability test, six replicates within one day of the sample were achieved, while for the inter-day variability test, duplicate measurements for consecutive three days were performed. Variations were expressed by the spike recoveries (RSDs) of the data. The spike recovery test was studied in order to examine the accuracy of the method evaluation. The recovery was performed by addition of a known amount of individual standards into a 1 mL well-distributed sample SQI-1 by shaking. Three replicates were performed for the recovery. The spike recoveries were evaluated with the following equation: Spike recovery (%) = (total amount detected − amount original)/amount spiked × 100%. The stability test was examined by testing the SQI-1 over a period of 2 h, 4 h, 6 h, 8 h, 12 h, and 24 h; stability measurements were represented by the RSDs of the peak areas of each analyte.

## 4. Conclusions

In this study, the special chemical profile of Shenqi Fuzheng (SQ) injection is qualitatively and quantitatively determined via a full component analysis approach. Up to 94.69% (*w*/*w*) of this injection product could be quantitatively determined, in which 15 determined non-saccharide small molecules and three determined monosaccharide/sucrose molecules account for 0.18%–0.21%, and 53.49%–58.2%, respectively. Additionally, adenosine contributes the highest dose of around 2 mg/day. No polysaccharides were found. The special composition is helpful to reach an explanation as to both the safety and therapy efficacy of SQI.

## Figures and Tables

**Table 1:** Identification of chemical constituents of SQ injection by UPLC-MS in positive and negative.

No.	RT (Min)	Formula	[M + H]^+^ (Error, ppm)	[M − H]^−^ (Error, ppm)	Fragment Ions in Positive Mode	Fragment Ions in Negative Mode	Identification
1	12.3	C_22_H_22_O_10_		445.1150 (3.59)		491.1215 [M + COOH]^−^, 481.0918 [M + Cl]^−^, 283.0623 [M − H − glc]^−^, 268.0375 [M − H − glc − H_2_O]^−^, 224.1435, 184.0517	Calycosin-7-*O*-beta-d-glucoside
2	14.9	C_9_H_16_O_4_		187.0976 (3.21)		169.0868, 125.0972	Azelaic acid
3	16.6	C_20_H_28_O_8_		395.1728 (5.82)		441.1773 [M + COOH]^−^, 431.1484 [M + Cl]^−^, 395.1701, 305.0586, 185.0970, 159.0812, 143.0708, 119.0351, 101.0243	Lobetyolin
4	19.2	C_23_H_28_O_10_		463.1619 (3.24)		509.1671 [M + COOH]^−^, 499.1383 [M + Cl]^−^, 345.9224, 301.1080, 254.0532, 135.0443	Isomucronulatol-7-*O*-glucoside
5	23.7	C_41_H_68_O_14_	785.4671 (2.04)	783.4554 (3.06)		(829.4593 [M + COOH]^−^, 819.4307 [M + Cl]^−^, 707.2920, 651.4105, 577.6834, 490.3592, 357.5564, 279.2332, 179.0567, 161.0450, 131.0343, 119.0352, 113.0245, 101.0246)	Astragaloside IV
6	23.8	C_41_H_68_O_14_		783.4538 (1.02)		(829.4593 [M + COOH]^−^, 819.4297 [M + Cl]^−^, 621.4005, 489.3581, 394.8312, 279.2329, 161.0464, 113.0251, 101.0243)	Astragaloside III
7	24.7	C_43_H_70_O_15_		825.4628 (0.97)		(871.4710 [M + COOH]^−^, 861.4408 [M + Cl]^−^, 765.4429, 719.7998, 520.4337, 338.1722, 224.1057, 179.0572, 143.0355, 119.0348, 101.0252)	Astragaloside II
8	25.2	C_48_H_78_O_18_	943.5269 (0.32)	941.5130 (2.23)		(879.5089, 733.4564, 615.3915, 457.3659, 362.4478, 247.0827, 163.0604)	Soyasaponin I
9	25.4	C_43_H_70_O_15_		825.4659 (2.79)		(871.4702 [M + COOH]^−^, 861.4404 [M + Cl]^−^, 726.3011, 593.3695, 465.2882, 336.2658, 257.2371, 179.0583, 113.0243)	Isoastragaloside II
10	26.0	C_43_H_70_O_15_		825.4618 (2.18)		(871.4705 [M + COOH]^−^, 861.4405 [M + Cl]^−^, 765.4444, 603.3852, 335.0594, 223.8866, 179.0537, 161.0435, 143.0337, 113.0220)	Cyclocephaloside II
11	1.8	C_10_H_13_N_5_O_4_	268.1059 (5.22)		211.9763, 178.0760, 136.0622, 119.0350		Adenosine
12	3.4	C_9_H_11_NO_2_	166.0863 (3.01)				l-Phenylalanine
13	5.1	C_14_H_21_NO_4_	268.1556 (2.98)		220.1272, 161.0537, 118.0810, 100.0709		Codonopsine
14	6.0	C_11_H_12_N_2_O_2_	[M + H − OH]^+^ 188.0943, (3.19)				Trytophan
15	17.1	C_22_H_22_O_9_	431.1343 (0.23)		453.1158 [M + Na]^+^, 350.0444, 269.0813, 213.0919, 118.0416		Ononin

**Table 2:** The calibration curves, linear ranges, limits of detection (LOD), limits of quantification (LOQ), precision, stability and accuracy of 18 analytes.

Analyte	EIC Ions	Linearity			LOD (µg/mL)	LOQ (µg/mL)	Repeatability RSD (%) (*n* = 6)		Stability	Spike Recovery (RSD %) (*n* = 3)		
		Regression Equation	*R*^2^	Range (µg/mL)			Intra-Day	Inter-Day	RSD (%) (*n* = 6)	High	Middle	Low
1	283.07	*y* = 85.094*x* − 47097	0.9997	3.04–12.16	0.15	0.49	1.61	3.74	3.12	101.00 (0.31)	104.61 (2.25)	95.32 (2.72)
2	187.10	*y* = 35.078*x* + 13967	0.9987	0.33–2.63	0.090	0.30	2.08	4.81	4.37	99.18 (3.01)	99.56 (5.04)	105.53 (1.83)
3	441.18	*y* = 31.521*x* − 15298	0.9981	1.22–3.80	0.14	0.47	2.03	2.27	3.18	97.11 (0.69)	103.34 (2.45)	96.33 (3.17)
4	463.17	*y* = 128.05*x* − 23035	0.9995	0.69–2.75	0.11	0.34	2.68	3.18	3.33	105.38 (0.71)	104.63 (0.13)	106.30 (0.53)
5	829.47	y = 595.32*x* + 39240	0.9992	0.50–4.00	0.0077	0.026	2.49	2.25	4.54	97.49 (0.80)	105.62 (0.63)	95.34 (2.88)
6	829.47	*y* = 453.52*x* − 9380.8	0.9991	0.19–0.93	0.0069	0.024	3.48	4.15	4.73	104.78 (1.25)	101.74 (3.98)	102.36 (3.40)
7	871.48	*y* = 410.39*x* − 220599	0.9990	0.57–2.85	0.11	0.36	1.32	1.33	1.48	100.62 (3.05)	105.35 (1.31)	95.35 (3.54)
8	941.52	*y* = 869.66*x* + 26058	0.9995	0.26–1.04	0.012	0.039	2.64	4.62	4.71	105.91 (5.70)	106.59 (2.95)	96.80 (3.58)
9	871.48	*y* = 393.52*x* + 9798.5	0.9992	0.52–2.60	0.14	0.49	1.69	2.90	3.88	94.97 (3.52)	98.60 (2.60)	105.16 (1.80)
10	871.48	*y* = 338.05x + 14751	0.9996	0.36–2.92	0.0048	0.016	1.59	2.39	2.75	102.57 (2.63)	95.38 (3.06)	96.58 (2.17)
11	268.11	*y* = 253.34*x* + 192461	0.9992	1.46–7.31	0.048	0.16	1.33	2.36	2.21	98.42 (1.79)	99.91 (0.37)	101.91 (1.91)
12	120.08	*y* = 159.12*x* + 104220	0.9991	1.48–7.38	0.102	0.34	1.38	2.45	2.16	98.82 (0.59)	99.90 (3.16)	98.75 (2.20)
13	268.16	*y* = 567.08*x* + 484891	0.9996	2.42–9.69	0.025	0.080	1.99	1.53	1.51	99.46 (0.68)	99.75 (0.57)	97.07 (6.77)
14	188.09	*y* = 1043.3*x* − 30639	0.9994	0.20–0.79	0.029	0.090	3.47	4.27	3.89	100.34 (0.94)	101.60 (1.20)	103.81 (4.25)
15	431.14	*y* = 125.32*x* − 18966	0.9996	0.87–2.24	0.17	0.56	3.74	4.33	4.31	103.98 (3.11)	99.77 (3.71)	105.11 (0.82)

**Table 3:** The contents (μg/mL) of 18 analytes in 13 SFI samples.

Analyte	SFI-1	SFI-2	SFI-3	SFI-4	SFI-5	SFI-6	SFI-7	SFI-8	SFI-9	SFI-10	SFI-11	SFI-12	SFI-13
1	7.53	7.60	7.52	7.60	7.69	7.90	7.25	7.22	7.46	7.45	5.67	5.83	5.63
2	0.36	0.42	0.43	0.38	0.41	0.44	0.38	0.35	0.38	0.32	1.04	1.28	1.27
3	2.48	2.71	2.79	2.76	2.66	2.63	2.58	2.16	2.50	2.48	1.94	2.81	2.46
4	1.80	1.89	1.86	1.86	1.91	1.91	1.89	1.84	1.85	1.80	1.64	1.86	1.84
5	2.61	2.95	2.81	2.90	2.83	2.88	2.83	2.83	2.85	2.83	2.12	2.32	2.29
6	0.44	0.49	0.45	0.47	0.46	0.45	0.46	0.45	0.45	0.45	0.34	0.31	0.31
7	2.26	2.61	2.43	2.50	2.50	2.51	2.48	2.48	2.49	2.52	1.98	1.91	1.90
8	1.31	1.37	1.40	1.38	1.39	1.28	1.35	1.38	1.31	1.33	0.93	1.06	1.04
9	0.97	1.21	1.10	1.12	1.10	1.12	1.14	1.08	1.13	1.14	0.77	0.78	0.72
10	1.12	1.44	1.30	1.31	1.30	1.30	1.26	1.32	1.38	1.37	0.94	0.95	0.87
11	7.39	7.82	7.92	7.64	7.42	7.23	7.38	7.31	8.04	8.18	7.06	8.48	8.60
12	5.52	5.82	5.77	5.70	5.74	5.47	5.36	5.46	5.15	5.55	5.49	5.32	5.25
13	5.10	4.99	5.02	4.78	4.79	4.84	5.16	4.89	4.93	4.92	6.10	4.93	4.87
14	0.33	0.30	0.27	0.36	0.31	0.29	0.30	0.37	0.26	0.26	0.19	0.31	0.43
15	1.92	1.82	1.73	1.77	1.86	1.71	1.32	1.63	1.85	1.76	1.17	1.18	1.15
Sub-total	40.56	42.94	42.20	41.99	41.79	41.52	40.65	40.26	41.59	43.00	37.06	38.93	38.23
Fructose ^a^	7.13	7.10	7.14	7.14	7.14	7.17	7.15	7.17	7.18	7.63	7.49	7.41	7.13
Glucose ^a^	2.72	2.73	2.73	2.84	2.82	2.76	2.75	2.78	2.73	3.04	2.76	2.74	2.72
Sucrose ^a^	1.33	1.33	1.33	1.37	1.33	1.34	1.35	1.36	1.34	1.22	1.23	1.21	1.33
Salt ^a,b^	7.2	7.2	7.2	7.2	7.2	7.2	7.2	7.2	7.2	7.2	7.2	7.2	7.2
**Dry weight** ^a^	19.91	20.39	19.91	20.16	19.57	19.79	19.59	20.12	20.02	20.43	20.97	20.59	20.9
**Content percentage**	92.52	90.25	92.63	92.22	94.69	93.54	94.39	92.20	92.37	93.65	89.26	90.33	88.13

^a^ The unit of weight was mg/mL; ^b^ The total content of NaCl, edetate disodium, and sodium pyrosulfite was set as 7.2 mg/mL as described in the QC standard of SFI [30,31,32].

## References

[1] Kwok K.Y., Xu J., Ho H.M., Chen H.B., Li M., Lang Y., Han Q.B. (2013). Quality evaluation of commercial Huang-Lian-Jie-Du-Tang based on simultaneous determination of fourteen major chemical constituents using high performance liquid chromatography. J. Pharm. Biomed. Anal..

[2] Zhang T.B., Yue R.Q., Xu J., Ho H.M., Ma D.L., Leung C.H., Chau S.L., Zhao Z.Z., Chen H.B., Han Q.B. (2015). Comprehensive quantitative analysis of Shuang-Huang-Lian oral liquid using UHPLC-Q-TOF-MS and HPLC-ELSD. J. Pharm. Biomed. Anal..

[3] Pan L. (2009). Practical road of “the numeral turn Chinese herbal medicine” for shenqi fuzheng injection. J. China Prescript Drug.

[4] Li H. (2012). Efficacy of shenqi fuzheng injection in adjuvant therapy of intractable heart failure. Chin. J. Geront..

[5] Yang J.Q., Li Y., Zhao Q., Kuang G., Fan L., Wang L., Zhang H., Jiao K., Zhou H. (2005). Effect of shenqi fuzheng injection on pre/post-operational change of argyrophilic-nucleolar organizer regions in peripheral T-lymphocyte in patients with gastric carcinoma. Chin. J. Intergr. Tradit. West Med..

[6] Zhao Q., Li Y., Wang L.L. (2001). Effect of shenqi fuzheng injection on immune function in gastric carcinoma patients in post-operational and chemotherapeutic period. Chin. J. Intergr. Tradit. West Med..

[7] Zhang Y., Guo L.L., Zhao S.P. (2010). Effect of shenqi fuzheng injection combined with chemotherapy in treating colorectal cancer. Chin. J. Intergr. Tradit. West Med..

[8] Lin H.S., Li D.R. (2007). Multi-center randomized clinical study on Shenqi Fuzheng injection combined with chemotherapy in the treatment for lung cancer. Chin. J. Oncol..

[9] Huang Z.F., Wei J.S., Li H.Z. (2008). Effect of shenqi fuzheng injection combined with chemotherapy on thirty patients with advanced breast cancer. Chin. J. Intergr. Tradit. West Med..

[10] Bo Y., Li H.S., Qi Y.C., Lu M.Y. (2007). Clinical study on treatment of mammary cancer by shenqi fuzheng injection in cooperation with chemotherapy. Chin. J. Integr. Med..

[11] Zhu X.Y., Zhang X.Z., Zhong X.Y. (2010). Effect of shenqi fuzheng injection for hemopoietic and immune function reconstruction in patients with hematologic malignancies undergoing chemotherapy. Chin. J. Intergr. Tradit. West Med..

[12] Li H.S., Yang B., Su X.C. (2009). Effect of shenqi fuzheng injection on repairing the immune function in patients with breast cancer. Chin. J. Intergr. Tradit. West Med..

[13] Liang Q.L., Pan D.C., Xie J.R. (2009). Effect of shenqi fuzheng injection combined with chemotherapy in treating advanced colorectal carcinoma. Chin. J. Intergr. Tradit. West Med..

[14] Dai Z., Wan X., Kang H., Ji Z., Liu L., Liu X., Song L., Min W., Ma X. (2008). Clinical effects of shenqi fuzheng injection in the neoadjuvant chemotherapy for local advanced breast cancer and the effects on T-lymphocyte subsets. J. Tradit. Chin. Med..

[15] Zhao J.M., Wu A.Z., Shi L.R. (2007). Clinical observation on treatment of advanced gastric cancer by combined use of shenqi fuzheng injection, docetaxel, flurouracil and calcium folinate. Chin. J. Intergr. Tradit. West Med..

[16] Li Z., Liu Z., Hou X., Qian Q., Liu S. (2002). Protective effect and mechanism of shenqi fuzheng injection on diabetic glomerulopathy in rats with streptozotocin-induced diabetes. Chin. J. Hosp. Pharm..

[17] Wang J., Tong X., Li P., Cao H., Su W. (2012). Immuno-enhancement effects of shenqi fuzheng Injection on cyclophosphamide-induced immunosuppression in Balb/c mice. J. Ethnopharm..

[18] Dong X.R., Wang J.N., Liu L., Chen X., Chen M.S., Chen J., Ren J.H., Li Q., Han J. (2010). Modulation of radiation-induced tumour necrosis factor-alpha and transforming growth factor beta1 expression in the lung tissue by shengqi fuzheng injection. Mol. Med. Rep..

[19] Chen J., Wang Z., Wu T. (2013). Shenqi fuzheng injection improves CVB3-induced myocarditis via inhibiting TRAF6 expression. Cell Mol. Biol..

[20] Song J.Z., Yiu H.W., Qiao C.F., Han Q.B., Xu H.X. (2008). Chemical comparison and classification of *Radix*
*astragali* by determination of isoflavonoids and astragalosides. J. Pharm. Biomed. Anal..

[21] Qiao C.F., He Z.D., Han Q.B., Xu H.X., Jiang R.W., Li S.L., Zhang Y.B., But P.P.H., Shaw P.C. (2007). The use of lobetyolin and HPLC-UV fingerprints for quality assessment of *Radix codonopsis*. J. Food Drug Anal..

[22] Napolitano A., Akay S., Mari A., Bedir E., Pizza C., Piacente S. (2013). An analytical approach based on ESI-MS, LC-MS and PCA for the quali-quantitative analysis of cycloartane derivatives in *Astragalus* spp.. J. Pharm. Biomed. Anal..

[23] Hu Q., Zhou T.S., Hu G., Fang Y.Z. (2002). Determination of sugars in Chinese traditional drugs by CE with amperometric detection. J. Pharm. Biomed. Anal..

[24] Lin L.C., Tsai T.H., Kuo C.L. (2013). Chemical constituents comparison of *Codonopsis tangshen*, *Codonopsis pilosula* var. *modesta* and *Codonopsis pilosula*. Nat. Prod. Res..

[25] Liu M.H., Tong X., Wang J.X., Zou W., Cao H., Su W.W. (2013). Rapid separation and identification of multiple constituents in traditional Chinese medicine formula shenqi fuzheng Injection by ultra-fast liquid chromatography combined with quadrupole-time-of-flight mass spectrometry. J. Pharm. Biomed. Anal..

[26] Kim E.Y., Kim J.A., Jeon H.J., Kim S., Kim Y.H., Kim H.Y., Whang W.K. (2014). Chemical fingerprinting of *Codonopsis pilosula* and simultaneous analysis of its major components by HPLC-UV. Arch. Pharm. Res..

[27] Zhang S., Fan C., Wang L., Liu X., Sun X., Ye W. (2011). Chemical constituents of shenqi fuzheng injection. Chin. Tradit. Pat. Med..

[28] Xu J., Chen H.B., Liu J., Kwok K.Y., Yue R.Q., Yi T., Ho H.M., Zhao Z.Z., Han Q.B. (2013). Why are *Angelicae Sinensis radix* and *Chuanxiong Rhizoma* different? An explanation from a chemical perspective. Food Res. Internat..

[29] Xu J., Li S.L., Yue R.Q., Ko C.H., Hu J.M., Liu J., Ho H.M., Yi T., Zhao Z.Z., Zhou J. (2014). A novel and rapid HPGPC-based strategy for quality control of saccharide-dominant herbal materials: *Dendrobium officinale*, a case study. Anal. Bioanal. Chem..

[30] Xu J., Yue R.Q., Liu J., Ho H.M., Yi T., Chen H.B., Han Q.B. (2014). Structural diversity requires individual optimization of ethanol concentration in precipitation of natural polysaccharides. Int. J. Biol. Macromol..

[31] Han X.Q., Chan B.C.L., Yu H., Yang Y.H., Hu S.Q., Ko C.H., Dong C.X., Wong C.K., Shaw P.C., Fung K.P. (2012). Structural characterization and immuno-modulating activities of a polysaccharide from *Ganoderma sinense*. Internat. J. Biol. Macromol..

[32] Livzon Pharm. Group Inc. (2005). A Immunomodulating Composition of Radix Codonopsis and Radix Astragali and its Preparation.

[33] Liu X., Tu F., Tao D. (2007). Quality Control of shenqi fuzheng Injections Containing *Radix codonopsis* and *Astragalus*.

[34] Pharmacopoeia Commission of P.R. China (2003). Formal Approved standard (s) for New Drugs.

[35] Li F., Cheng T.F., Dong X., Li P., Yang H. (2016). Global analysis of chemical constituents in shengmai injection using high performance liquid chromatography coupled with tandem mass spectrometry. J. Pharm. Biomed. Anal..

[36] Yang H., Liu L., Gao W., Liu K., Qi L.W., Li P. (2014). Direct and comprehensive analysis of ginsenosides and diterpene alkaloids in Shenfu injection by combinatory liquid chromatography-mass spectrometric techniques. J. Pharm. Biomed. Anal..

